# *Lycium barbarum* Oligosaccharides Alleviate Hepatic Steatosis by Modulating Gut Microbiota in C57BL/6J Mice Fed a High-Fat Diet

**DOI:** 10.3390/foods12081617

**Published:** 2023-04-11

**Authors:** Mengjie Li, Zheng Zhang, Bin Yu, Siqiang Jia, Bo Cui

**Affiliations:** 1 State Key Laboratory of Biobased Material and Green Papermaking, Qilu University of Technology, Shandong Academy of Sciences, Jinan 250353, China; limj5388@163.com (M.L.); zhengzhang324@163.com (Z.Z.); yubin@qlu.edu.cn (B.Y.); jiasq0001@163.com (S.J.); 2 School of Food Science and Engineering, Qilu University of Technology, Shandong Academy of Sciences, Daxue Road, Changqing District, Jinan 250353, China

**Keywords:** *Lycium barbarum* oligosaccharides, high-fat diet, gut microbiota, fecal metabolites, hepatic steatosis

## Abstract

High-fat diets (HFD) can promote the development of hepatic steatosis by altering the structure and composition of gut flora. In this study, the potential therapeutic mechanism of *Lycium barbarum* oligosaccharide (LBO) against hepatic steatosis was investigated by analyzing the changes in the intestinal flora and metabolites in mice. Mice on an HFD were administered LBO by gavage once daily for a continuous period of eight weeks. Compared with the HFD group, the levels of triglyceride (TG), alanine aminotransferase (ALT) in the serum, and hepatic TG were significantly reduced in the LBO group, and liver lipid accumulation was obviously improved. In addition, LBO could regulate the HFD-induced alteration of intestinal flora. The HFD increased the proportion of *Barnesiellaceae*, *Barnesiella*, and *CHKCI001*. LBO increased the proportion of *Dubosiella*, *Eubacterium*, and *Lactobacillus*. LBO also altered the fecal metabolic profile. Significantly different metabolites between LBO and the HFD, such as taurochenodeoxycholate, taurocholate, fluvastatin, and kynurenic acid, were related to the cholesterol metabolism, bile acid metabolism, and tryptophan metabolic pathways. In light of the above, LBO can alleviate HFD-induced NAFLD by modulating the components of the intestinal flora and fecal metabolites.

## 1. Introduction

Nonalcoholic fatty liver disease (NAFLD) is a metabolism-related hepatic disease, and its morbidity and mortality have rapidly increased in recent years [1]. The characteristic feature of NAFLD is an excess deposition of triglycerides (TGs) in the liver devoid of alcohol abuse or other precipitating factors, such as genetic disorders [2]. Pathologically, nonalcoholic fatty liver (NAFL) and nonalcoholic steatohepatitis (NASH) are two different diseases of NAFLD. The latter comprises a broad range from fibrosis to cirrhosis and ultimately to liver failure and hepatocellular carcinoma. NAFLD, the most ordinary type of hepatic disease in Western countries, has a prevalence of 17–46%, with differences in regard to diagnostic methods, age, sex, and race [3]. A high-sugar, high-calorie diet causes the caloric intake to be too high and energy metabolism to be unbalanced, which is one of the important factors in the formation of NAFLD [4]. Currently, lifestyle and pharmacological treatment are two important treatment measures for NAFLD [5]. Nevertheless, there are no clear drug treatment options for NAFLD [1]. Consequently, there is a need for safer and more efficacious strategies, such as those involving natural functional compounds, to prevent the development of NAFLD.

*Lycium barbarum* is a traditional Chinese medicinal and edible plant that can lower blood sugar and blood lipids and provide good protection for the liver [6]. The *L. barbarum* polysaccharide (LBP) is an important active substance in the fruits of *L. barbarum*, and has effects on regulating lipid metabolism and improving hepatic steatosis [7,8]. However, LBP has a complex structure and poor water solubility because it comprises highly branched polysaccharides and proteins [9]. The oligosaccharides produced by polysaccharide hydrolysis have low molecular weight and enhanced water solubility [10]. A previous study found that the *L. barbarum* oligosaccharide (LBO) improves type 2 diabetes. Moreover, LBO can alleviate liver injury by restoring the morphology of hepatocytes and improving hepatic steatosis and inflammation [11]. The oligosaccharides present in *L. barbarum* may be potential prebiotics that exhibit hypolipidemic activity, such as lowering total cholesterol levels [12]. Prebiotics are substrates that can be selectively utilized by host gut microbes and have beneficial effects on health. They have an impact on beneficial microorganisms and produce metabolites that are beneficial to health [13]. Therefore, prebiotics can change intestinal permeability, regulate the composition of gut microbiota, and relieve liver damage via the gut–liver axis, thus affecting the process of NAFLD [14,15]. From this, we can speculate that LBO plays a part in alleviating hepatic steatosis. Nevertheless, the mechanism by which LBO alleviates hepatic steatosis still needs to be further explored.

In the body, the first organ affected by diets is the gastrointestinal tract [16]. The gut is a complex ecosystem comprising hundreds of millions of microorganisms. The thriving of microorganisms is closely associated with health and disease. The intestinal flora can regulate the effects of diets on the metabolic mechanisms of the body and have potential therapeutic effects on diseases [17]. In a normal physiological state, the gut microbiota of an organism maintain a dynamic balance. When diseases such as fatty liver, obesity, and type 2 diabetes develop, this balance is disrupted, and structural and functional disorders of the intestinal flora occur [18]. It has been found that an imbalance of intestinal flora can be regulated by antibiotics, prebiotics, probiotics, and fecal microbial transplants. Among them, the regulation of intestinal dysbiosis by prebiotics is a safer and more effective treatment [19]. 

The purpose of this research was to examine LBO in the role of alleviating the NAFLD process in HFD-treated mice and analyze the underlying mechanisms. We hypothesized that LBO could alleviate NAFLD by altering the structure of the intestinal flora and its metabolites, and the corroboration of this result will be helpful to determine alternative strategies to alleviate HFD-induced hepatic steatosis and liver injury.

## 2. Materials and Methods 

### 2.1. Chemical Characterization of LBO 

The LBP was extracted by referring to the method of Ding et al. [20]. First of all, the ground powder of dried *L. barbarum* fruits was extracted with water and then precipitated with ethanol. After the protein was removed with the Sevag reagent (CHCl_3_:n-BuOH = 4:1), the solution was dialyzed and lyophilized to obtain LBP. LBO was extracted from LBP by referring to the method of Liu et al. [11]. Pronase E (Solarbio Technology Co., LTD, Beijing, China), H_2_SO_4_, and trifluoroacetic acid (TFA) were used to degrade *L. barbarum* polysaccharide, which then underwent desalting and concentration to obtain LBO. The dried LBO samples were pressed with Kbr powder to form clear, pressed sheets. Fourier transform infrared (FT-IR) spectroscopy was measured in 4000~400 cm^−1^ using a FT-IR spectrometer (Perkin Elmer, Waltham, MA, USA). Mannose (Man), glucosamine (GlcN), ribose (Rib), rhamnose (Rha), glucuronic acid (GlcUA), galacturonic acid (GalA), aminogalactose (GalN), glucose (Glu), galactose (Gal), xylose (Xyl), arabinose (Ara), and fucose (Fuc) were used as standards for the identification of the monosaccharide fractions of LBO by ion chromatography (IC, DIONEX, Sunnyvale, CA, USA). The column was CarboPac PA20 (3 mm × 150 nm) and the mobile phase was H_2_O, NaOH (250 mmol/L), and NaAc (1 mol/L). High performance gel permeation chromatography (HPGPC, Agilent, Santa Clara, CA, USA) with a 2410 differential refractive index detector and an Empower workstation was used to analyze the molecular weight distribution of oligosaccharides. The chromatographic column was Ultrahydrogel^TM^ Linear (330 mm × 7.8 mm) and the mobile phase was 0.1 N sodium nitrate.

### 2.2. LBO In Vitro Simulated Digestion 

#### 2.2.1. Reduced Sugar Content Determination 

Glucose solutions of gradient concentrations (0, 0.2, 0.4, 0.6, 0.8, and 1 mg/mL) were prepared. A total of 1 mL of glucose solutions at different concentrations was mixed with 2 mL of 3, 5-dinitrosalicylic acid (DNS) solution (Solarbio Technology Co., Ltd., Beijing, China), followed by a boiling water bath for 15 min. After cooling to room temperature, 9 mL of distilled water was added, and the OD value was measured at 540 nm using a UV spectrophotometer (TU-1810, Beijing Persee General Instrument Co., Ltd., Beijing, China). The standard curve was plotted according to the glucose concentration and the corresponding OD value. 

#### 2.2.2. Total Sugar Content Determination 

A standard solution of glucose in a concentration gradient of 0.01 mg/mL–0.08 mg/mL was prepared. In total, 1 mL of the glucose solution was mixed with 1.6 mL of the 5% phenol solution and 7 mL of concentrated H_2_SO_4_. The OD value was measured at 490 nm using a UV spectrophotometer after 25 min. The standard curve was drawn according to the glucose concentration and the corresponding OD value. In total, 1 mL of the LBO solution was taken in a test tube and the absorbance was measured according to the standard curve method. For the blank control, distilled water was used instead of samples.

#### 2.2.3. In Vitro Digestion Simulation 

Gastric and small intestinal media were configured with reference to the method of Hu et al. [21]. To simulate gastric digestion, 4 mL of LBO solution and 10 mL of gastric medium were taken and placed in a test tube. The tubes were incubated in a shaker (37 °C, 200 rmp). Then, 1 mL of the digest was taken at 0, 2, 4, and 6 h, respectively, and placed immediately in boiling water for 5 min. The absorbance of the digest was measured according to the above standard curve plotting method. 

After 6 h of digestion in a gastric medium, the pH of the digest in the tubes was adjusted to 7 with NaHCO_3_, and then 1.8 mL of a small intestine medium was added and incubated in a shaker under the same conditions. In total, a 1 mL mixture was collected at 0, 2, 4, and 6 h, and then immersed instantly into a boiling water bath for 5 min. According to the above standard curve plotting method, the absorbance was measured.

#### 2.2.4. Hydrolysis Degree Determination of LBO

After measuring the reducing sugar content and the total sugar content, the degree of LBO hydrolysis was calculated according to the following formula.

$$R\left(\%\right)=\frac{C_{1}-C_{0}}{C_{2}}\times 100$$

In the formula, R represents the degree of hydrolysis, C_0_ represents the reducing sugar content before hydrolysis, C_1_ represents the reducing sugar content after hydrolysis, and C_2_ represents the total sugar content. 

### 2.3. Animal Feeding and Experimental Design 

Eight-week-old male C57BL/6J mice were purchased from Pengyue Biotechnology Co., Ltd. (Jinan, China) and housed in cages with temperature (25 ± 2 °C) and humidity (60% ± 10%) controlled by a 12 h light/12 h dark cycle in the Laboratory Animal Room. Water and food were freely available to all mice. At the end of the seven days of acclimatization feeding, eighteen mice were haphazardly allocated into three groups: the control diet group (Control, n = 6), HFD group (HFD, n = 6), and treatment group (LBO, n = 6). The control mice were fed with normal chow (D12450B diet, 10% fat); mice in the HFD and LBO groups were fed the HFD diet (D12451 plus 2% cholesterol diet, 47% fat) for eight weeks (Sun et al., 2019). Mice in LBO were administered LBO (200 mg/kg body weight) mixed in normal saline (NS) by daily oral gavage [11]. Other groups were gavaged with an equal volume of NS. The weekly body weight and daily food intake of mice were documented. After 8 weeks, fresh mouse feces were collected with sterile cryotubes and immediately frozen in liquid nitrogen, and then stored at −80 °C for subsequent fecal flora sequencing and fecal metabolite analysis.

At the end of the experiment, mice were sacrificed by cervical dislocation after fasting for 12 h. Blood was collected from the eye socket using orbital extraction and centrifuged (1000× *g*, 4 °C) for 10 min. Serum samples were collected. The liver was harvested. All steps of this experiment were approved by the Animals Ethics Committee of the Experimental Animal Centre of Shandong University of Traditional Chinese Medicine (No. SYXKLU20170022, Jinan, China), and research was conducted consistently with the requirements of EU Directive 2010/63/EU.

### 2.4. Serum and Hepatic Biochemical Analysis 

Biochemical indicators such as alanine aminotransferase (ALT), alkaline phosphatase (ALP), total cholesterol (TC), and triglycerides (TGs) in serum were measured by an automatic chemistry analyzer (Hitachi, 7600P, Tokyo, Japan). The levels of serum total superoxide dismutase (T-SOD) were measured using ELISA kits (Nanjing Jiancheng Bioengineering Engineering Research Institute, Nanjing, China) in accordance with the instructions. The contents of TG, TC, and T-SOD were also measured by this method. 

### 2.5. Histological Staining 

After rinsing with saline, the largest lobe of liver tissue was fixed in a 10% neutral buffered formalin solution. The liver tissue was embedded in paraffin, sectioned, and stained with a hematoxylin-eosin (H&E) stain. The structural changes in liver tissues were observed under light microscopy (Olympus, Tokyo, Japan). To detect hepatic steatosis by Oil Red O staining, hepatic tissues were sectioned using a frozen sectioning machine. After rewarming and drying the frozen sections, they were fixed with a fixing solution and stained using Oil Red O for 10 min at 37 °C. Hematoxylin was used to stain the sections. Finally, they were observed under a microscope. The NAFLD activity scoring system (NAS) was used to assess histological severity [22].

### 2.6. Fecal Microbiota Sequencing and Bioinformatic Analysis 

The approach applied to extract the total genomic DNA from the fecal samples of mice was the hexadecyltrimethylammonium bromide method. HiFi HotStart ReadyMix (KAPA Biosystems, Woburn. MA, USA) coupled with the barcoded primers 341F (5′-CCTACGGGAGGCAGCAG-3′) and 806R(5′-GGACTACHVGGGTWTCTAAT-3′) can be used to amplify the V3-V4 variable regions of the 16S rRNA gene. Mixed PCR products were purified using the AxyPrepDNA Gel Extraction Kit (Axygen Biosciences, Union City, CA, USA). After quantification, equivalent amplicons were sequenced using an Illumina MiSeq/HiSeq2500 platform. 

The paired-end reads were combined with raw DNA fragments using FLASH. Unique barcodes were utilized to distribute paired-end reads to every sample. Sequences with ≥97% similarity were grouped into identical OTUs. The unweighted UniFracBeta distance was applied to a principal coordinate analysis (PCoA), and the unweighted pair group method with arithmetic mean (UPGMA) clustering, complex, and multidimensional data was obtained and visualized in master coordinates by the PCoA. A variation ranging from the distance matrix to a new set of orthogonal axes is required for this type of analysis. The first principal coordinate was used to denote the maximum variation factor, the second principal coordinate was used to denote the second maximum variation factor, and so on. UPGMA clustering, a hierarchical clustering method using average linkage, can be applied to explain the distance matrix. Discrepancies in the taxonomic abundance of individuals in the two groups were confirmed using STAMP software. Biomarkers were quantified among three groups with the linear discriminant analysis (LDA) effect size algorithm (LEfSe).

### 2.7. Metabolomics Data Analysis 

Metabolites in fecal samples were detected by an ultra-high-performance liquid chromatography-quadrupole time-of-flight mass spectrometer (UHPLC-Q-TOF/MS). To facilitate extraction, the samples were thawed slowly at 4 °C, and fecal samples were added to the prechilled methanol/acetonitrile/water solution (2:2:1, *v/v*). After mixing well, the samples were first hatched on ice for 20 min and then centrifuged (14,000× *g*, 4 °C) for 20 min; finally, the supernatant was desiccated under a vacuum. The mass spectrometry required the addition of 100 μL of acetonitrile (acetonitrile:water = 1:1, *v/v*), vortexing, and centrifugation at 14,000× g and 4 °C for 15 min. The samples were separated by a UHPLC system (Agilent 1290 Infinity LC) coupled with a hydrophilic interaction chromatography (HILIC) column (Agilent Technologies, Santa Clara, CA, USA). The column temperature was 25 °C, the flow rate was 0.5 mL/min, and the injection volume was 2 μL. After sample separation, a Triple TOF 6600 MS (AB SCIEX, Framingham, MA, USA) with electrospray ionization (ESI) was applied to analyze the samples in positive and negative ion modes for mass spectrometry. After the original data were transformed into mzXML format using ProteoWizard, peak alignment, retention time correction, and peak area extraction were carried out by XCMS software. The XCMS-extracted data were initially applied for metabolite structure determination, data preprocessing, and, later, data quality assessment.

### 2.8. Statistical Analyses 

All values are represented as the mean ± standard deviation (SD). SPSS 22.0 software (Chicago, IL, USA; version 22.0) was applied to the *t*-tests. R statistical software (Foundation for Statistical Computing, Vienna, Austria; version 3.4.0) was used for the analyses. Significance was indicated by *p* < 0.05. The SIMCA 14.1 software wrapper (MKS Data Analytics Solutions, Umea, Sweden) was applied to multivariate analyses. 

## 3. Results 

### 3.1. Chemical Characterization of LBO 

**Figure 1:**
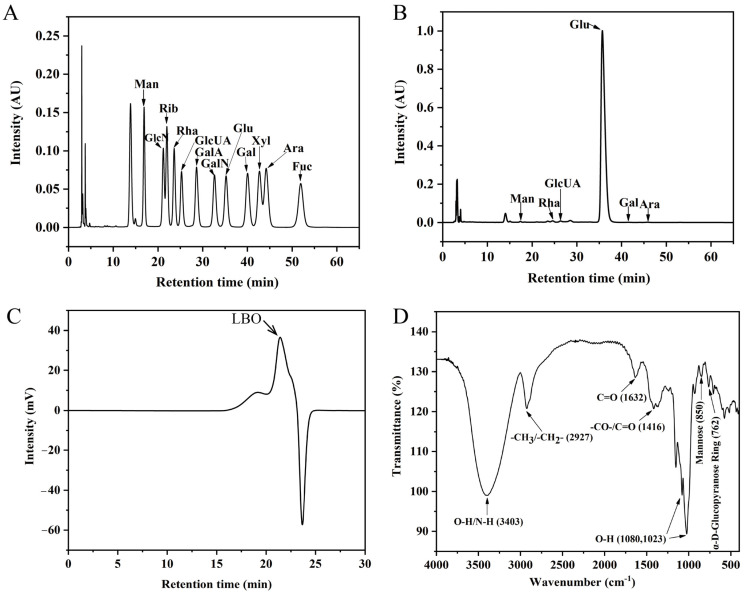
Chemical characterization of LBO. (**A**) IC of standard monosaccharides. (**B**) IC of LBO. (**C**) HPGPC of LBO. (**D**) FT-IR chromatogram of LBO. LBO: *Lycium barbarum* oligosaccharide; IC: ion chromatography; HPGPC: high performance gel permeation chromatography; FT-IR: Fourier transform infrared.; Man: mannose; GlcN: glucosamine; Rib: ribose; Rha: rhamnose; GlcUA: glucuronic acid; GalA: galacturonic acid; GalN: aminogalactose; Glu: glucose; Gal: galactose; Xyl: xylose; Ara: arabinose; Fuc: fucose.

The IC showed that LBO was composed of Rha, Man, Glu, GlcUA, Gal, and Ara (Figure 1A,B). The HPGPC indicated an obvious peak at 22.416 and the molecular weight of LBO was around 880 Da (Figure 1C). Based on the above results, it can be deduced that LBO is an oligosaccharide consisting of six monosaccharides with a molecular weight of around 880 Da. The result of the FT-IR spectral analysis of the LBO is shown in Figure 1D [11].

### 3.2. Changes in the Degree of Hydrolysis of LBO during Simulated Digestion 

As shown in Table 1, the degree of hydrolysis of LBO during simulated gastrointestinal digestion was almost 0. It is clear that LBO cannot be digested in the intestine. Therefore, LBO is a functional oligosaccharide that can be used as a prebiotic to balance intestinal flora.

### 3.3. Changes in Body Weight and Food Intake in Mice 

**Figure 2:**
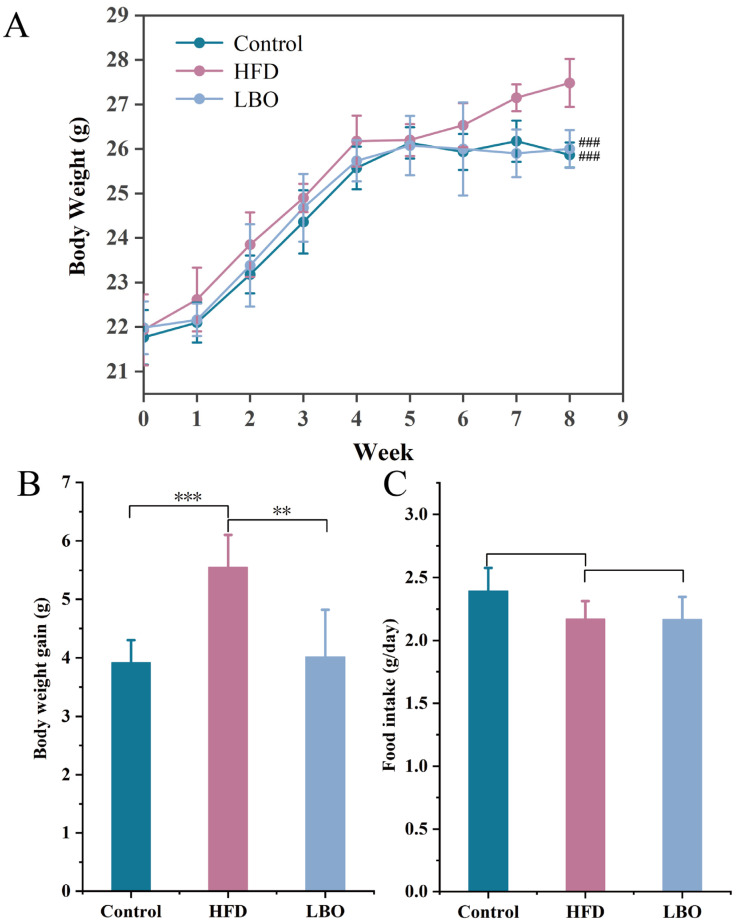
Ability of LBO to affect body weight changes in HFD-fed mice. (**A**) Change in the body weight of mice after eight weeks of housing. ^###^ *p* < 0.001 vs. the HFD group. (**B**) Amount of body weight gain in mice. (**C**) Daily food intake of mice. Figures are presented as the mean ± SD, n = 6. ** *p* < 0.01, *** *p* < 0.001. LBO: *Lycium barbarum* oligosaccharide; HFD: high-fat diet.

To explore the alleviating effect of LBO on HFD-induced hepatic steatosis and liver injury, LBO was administered to HFD-fed C57BL/6J mice for eight consecutive weeks. Compared with the control group, mice in the HFD group showed a significant increase in final body weight (Figure 2A), while the addition of LBO significantly reduced the body weight gain (BWG) caused by the HFD (Figure 2B). Notably, there was no obvious change in the average daily food intake among the three groups (Figure 2C). The above findings suggested that LBO can reduce the BWG caused by HFD without affecting the appetite of mice. 

### 3.4. Effects of LBO on Hepatic Steatosis and Liver Injury in Mice 

**Figure 3:**
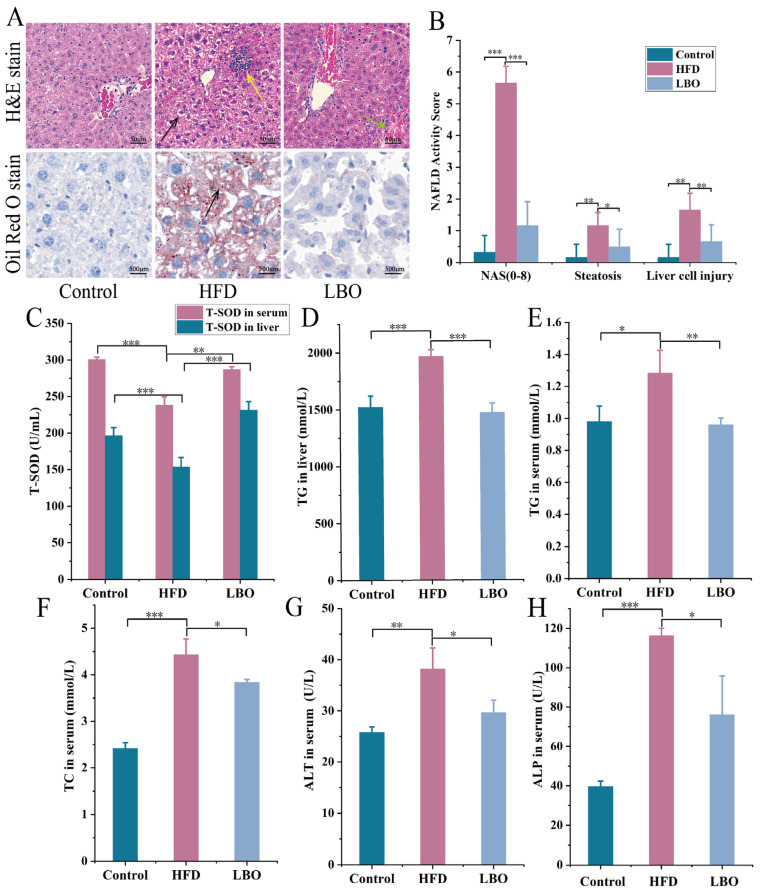
Alleviating efficacy of LBO on hepatic injury in HFD mice. (**A**) H&E staining of the liver (200×) and Oil Red O staining. In H&E staining, black arrows denote swollen liver cells with vacuolated cytoplasm, and yellow arrows reveal inflammatory cell infiltration. In Oil Red O staining, the black arrows represent lipid droplets in the liver. (**B**) NAFLD activity scores. (**C**) The level of T-SOD in liver and serum. (**D**) TG levels in serum, (**E**) TG levels in the liver, (**F**) serum TC level, (**G**) ALT level, and (**H**) ALP level. Values are presented as the mean ± SD, n = 6. * *p* < 0.05, ** *p* < 0.01, *** *p* < 0.001. LBO: *Lycium barbarum* oligosaccharide; HFD: high-fat diet; NAFLD: nonalcoholic fatty liver disease; T-SOD: total superoxide dismutase; TG: triglycerides; TC: total cholesterol; ALT: alanine aminotransferase; ALP: alkaline phosphatase.

The morphology of the liver tissue was evaluated using H&E staining and Oil Red O staining. In the control group, the hepatic tissue structure was normal with intact hepatocyte morphology, clear nuclei, and abundant cytoplasm. In addition, no edema or steatosis was observed, and there was no significant inflammatory cell infiltration. For the HFD group, as shown in Figure 3A, the morphology of the liver tissue was severely abnormal, and some of the hepatocytes were heavily edematous with swollen cells and vacuolated cytoplasm (black arrows); a mass of inflammatory cells was also seen infiltrating the liver parenchyma (yellow arrows). The addition of LBO significantly improved the effect of an HFD on the liver tissue structure. The LBO group had a mild abnormal liver tissue structure, and some liver sinuses were mildly dilated and congested (Figure 3A). LBO was observed to alleviate liver inflammation and have a protective effect on hepatocytes. Oil Red O staining showed many lipid droplets visible in the liver of the HFD group (black arrow), and the addition of LBO greatly reduced the number of hepatic lipid droplets (Figure 3A). Based on H&E and Oil Red O staining, the histological scoring for steatosis and hepatocyte injury showed that LBO significantly improved HFD-induced liver injury and steatosis (Figure 3B). As expected, oral LBO significantly inhibited hepatic lipid deposition. 

Figure 3 shows the serum and hepatic biochemical indices of the three groups after 8 weeks of LBO supplementation. T-SOD and TG levels in the liver were measured (Figure 3C,D). The results showed that LBO significantly reduced the TG content in liver tissue (*p* < 0.05), but there was no significant change in hepatic TC content (*p* > 0.05; data not shown). The T-SOD concentration was markedly increased in the LBO diet in comparison with the HFD (*p* < 0.05). Additionally, LBO supplementation significantly ameliorated the abnormal serum metabolic profile, including T-SOD (Figure 3C), TG (Figure 3E), TC (Figure 3F), ALT (Figure 3G), and ALP (Figure 3H) in the serum. Serum TG, TC, ALT, and ALP contents in the LBO group were appreciably decreased, and T-SOD levels were obviously higher than in the HFD group (*p* < 0.05). The level of ALT, a clinical marker to determine liver injury (Harrison et al., 2008), decreased significantly after oral LBO, indicating that LBO can reduce hepatocellular injury and has a protective effect on the liver [23]. 

### 3.5. Effects of LBO Supplementation on Fecal Microbiota 

**Figure 4:**
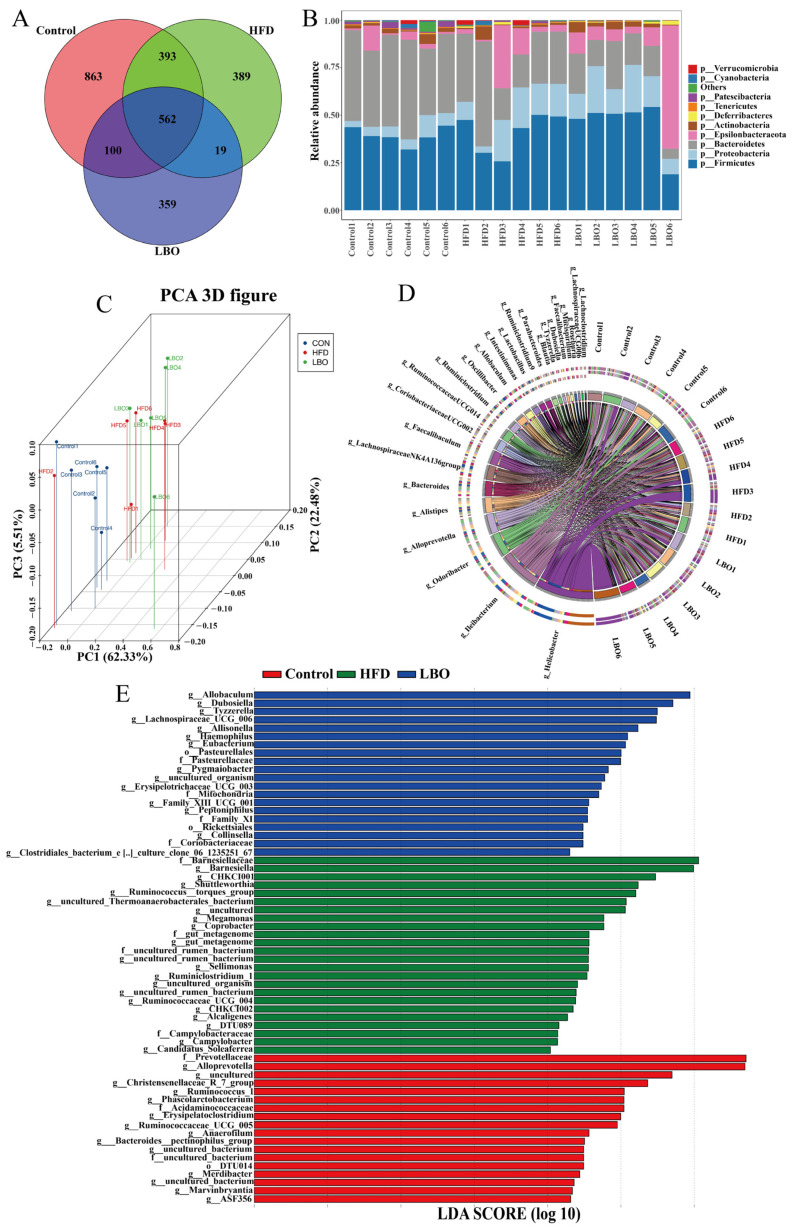
Effect of LBO on the structure and form of the gut microbiota in HFD mice. (**A**) Venn diagram. Different colors represent different groups. The overlapping areas of different colored circles indicate that the different groups were divided into the same OTU. The nonoverlapping parts of different colored circles indicate that each group had a sequence belonging to a different OTU. (**B**) Relative abundance of major microorganisms in the three groups at the phylum level. (**C**) Isolation of fecal microbial structure expressed by PCoA in the three groups. (**D**) The 25 most abundant genera in the different groups. (**E**) Linear discriminant analysis (LDA) effect size (LEfSe) analyses of the three groups at the genus level. LBO: *Lycium barbarum* oligosaccharide; HFD: high-fat diet; PCoA: principal coordinate analysis.

To investigate the effect of LBO on the intestinal flora, we conducted 16S rRNA high throughput sequencing of mouse feces. After filtering out ineligible sequences, the remaining effective tags in the control, HFD, and LBO groups were 65,279, 67,016, and 64,280, respectively. Representative sequences of OTUs were species annotated. The Venn diagram indicated that there were 863 unique OTUs in the control group, 389 unique OTUs in the HFD group, 359 unique OTUs in the LBO group, and a total of 562 OTUs for the three groups (Figure 4A). The PCoA showed an obvious separation of intestinal flora structure among the three groups. PC1, PC2, and PC3 accounted for 62.33%, 22.48%, and 5.51% of the total variance (Figure 4C), respectively, indicating the consistency and reliability of the model. The composition of the flora was significantly and independently aggregated, suggesting that LBO had a remarkable effect on the composition of the intestinal flora. 

By analyzing the composition of the enteric microorganism community at the phylum and genus levels, we further evaluated the overall structure of the bacterial communities of different taxa. The relative species abundance histogram at the phylum level revealed that the mouse gut microbiota all belonged to 10 phyla: Verrucomicrobia, Cyanobacteria, Patescibacteria, Tenericutes, Deferribacteres, Actinobacteria, Epsilonbacteraeota, Bacteroidetes, Proteobacteria, and Firmicutes. In comparison with the HFD group, the addition of LBO enhanced the proportion of Actinobacteria and Firmicutes and decreased the proportion of Bacteroidetes (Figure 4B). The ratio of Firmicutes/Bacteroidetes increased in the LBO group. At the genus level, the addition of LBO increased the relative abundance of *Alloprevotella* and *Lactobacillus* (Figure 4D). LEfSe was further used to analyze and compare the intestinal flora and showed significant differences in abundance at the genus level of the three taxa. The dominant flora in the control group at the genus level included *Alloprevotella*, *Ruminococcus_1*, *Phascolarctobacterium*, *Erysipelatoclostridium*, *Ruminococcaceae_UCG_005*, and *Anaerofilum*. In the HFD group, *Barnesiellaceae*, *Barnesiella*, *CHKCI001*, *Shuttleworthia*, *Megamonas*, and *Coprobacter* were more abundant as biomarkers, while the LBO group primarily showed a higher enrichment of *Allobaculum, Dubosiella*, *Tyzzerella*, *Lachnospiraceae_UCG_006*, *Allisonella*, and *Eubacterium* as biomarkers (LDA score > 2.0 with *p* < 0.05) (Figure 4E). Our findings indicated that LBO is capable of regulating the composition of intestinal flora. 

### 3.6. Effects of LBO Addition on Fecal Metabolite Profiles

**Figure 5:**
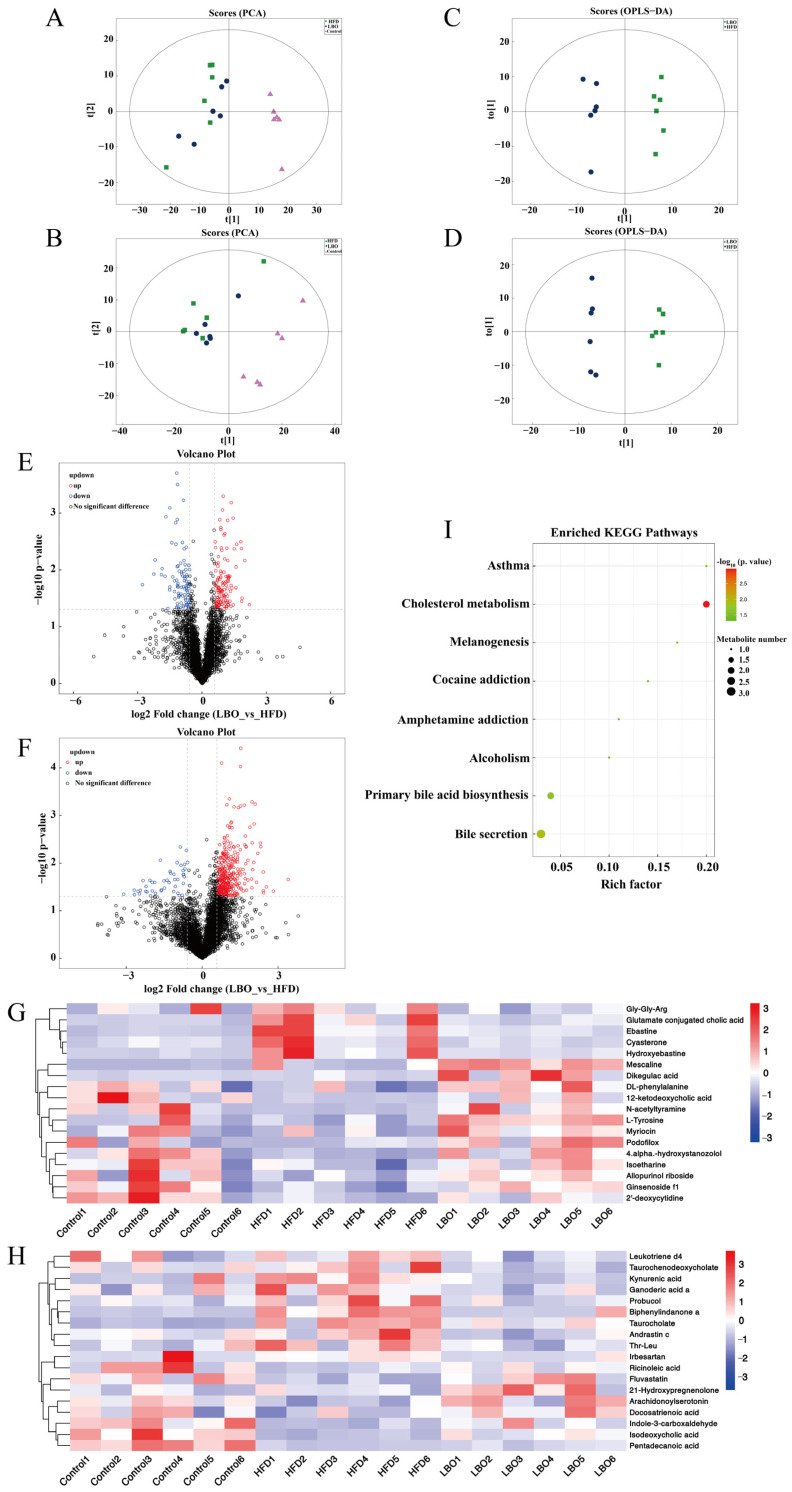
Effect of LBO on fecal metabolites in HFD mice. The PCA scatter diagrams in (**A**) negative and (**B**) positive ion modes. Plots of OPLA-DA scores in (**C**) negative and (**D**) positive ion modes. Significant differences in metabolites between HFD and LBO were indicated by a volcano plot. Differential metabolites in (**E**) negative and (**F**) positive ion modes. The hierarchical clustering heatmap showed (**G**) metabolites with significant differences in the negative ion mode (*VIP* > 2 and *p* < 0.05) and (**H**) metabolites with significant differences in the positive ion mode (*VIP* > 1 and *p* < 0.05). (**I**) Analysis of the KEGG metabolic pathway between HFD and LBO. Each metabolic pathway is represented by a bubble in the diagram. The size of the air bubbles represents the quantity of metabolites. The *p* value (the negative common logarithm, −log10 *p* value) of the enrichment analysis is expressed by the color of the bubbles; the darker the color, the lower the *p* value and the more significantly enriched. LBO: *Lycium barbarum* oligosaccharide; HFD: high-fat diet; PCA: principal component analysis; *VIP*: variable importance in the projection.

Fecal metabolomics, as a function of microbiome reading, explain the impact of gut microbes on host health and establish a link between the host, diet, and gut microbes [24]. In total, 2605 metabolites were evaluated in positive and negative binding modes, among which 1659 metabolites were found in the positive ion mode and 946 metabolites were found in the negative ion mode. The principal component analysis (PCA) score results indicated that there was a separation of metabolites among the three groups (Figure 5A,B). Volcano plots were used to represent differential metabolite changes between the LBO and HFD groups in positive and negative ion patterns (Figure 5E,F). The volcano plots expressed one metabolite per dot, with red dots denoting the upregulated metabolites, blue dots denoting the downregulated metabolites, and black dots indicating the metabolites that were not significantly different. However, the volcano map contained a large number of variables that did not allow the visualization of changes in specific differential metabolites. Therefore, the variable importance in the projection (VIP) value (>1.0) of the orthogonal partial least squares discriminant analysis and the *p* value (<0.05) of Student’s *t*-test were used to identify 36 differential metabolites with respect to the HFD and LBO groups (Figure 5G,H). Then, heatmap visualization was applied to further detail the analyses of differential metabolites. As observed in the figure, the metabolites in the LBO group were upregulated, including fluvastatin, ricinoleic acid, 21-hydroxypregnenolone, indole-3-carboxaldehyde, isoetharine, allopurinol riboside, and DL-phenylalanine. Furthermore, the abundance of metabolites such as taurochenodeoxycholate, kynurenic acid, and pentadecanoic acid significantly decreased (Figure 5G,H). An analysis of biochemical pathways in the KEGG database and remarkable differences in metabolites between the HFD and LBO groups indicated that the major metabolic pathways included bile acid secretion, primary bile acid biosynthesis, and cholesterol metabolism (Figure 5I). From the above data, it is clear that an HFD causes a disturbance of intestinal metabolites, and that oral LBO can restore intestinal homeostasis.

### 3.7. Analysis of the Association between Fecal Flora and Metabolites 

**Figure 6:**
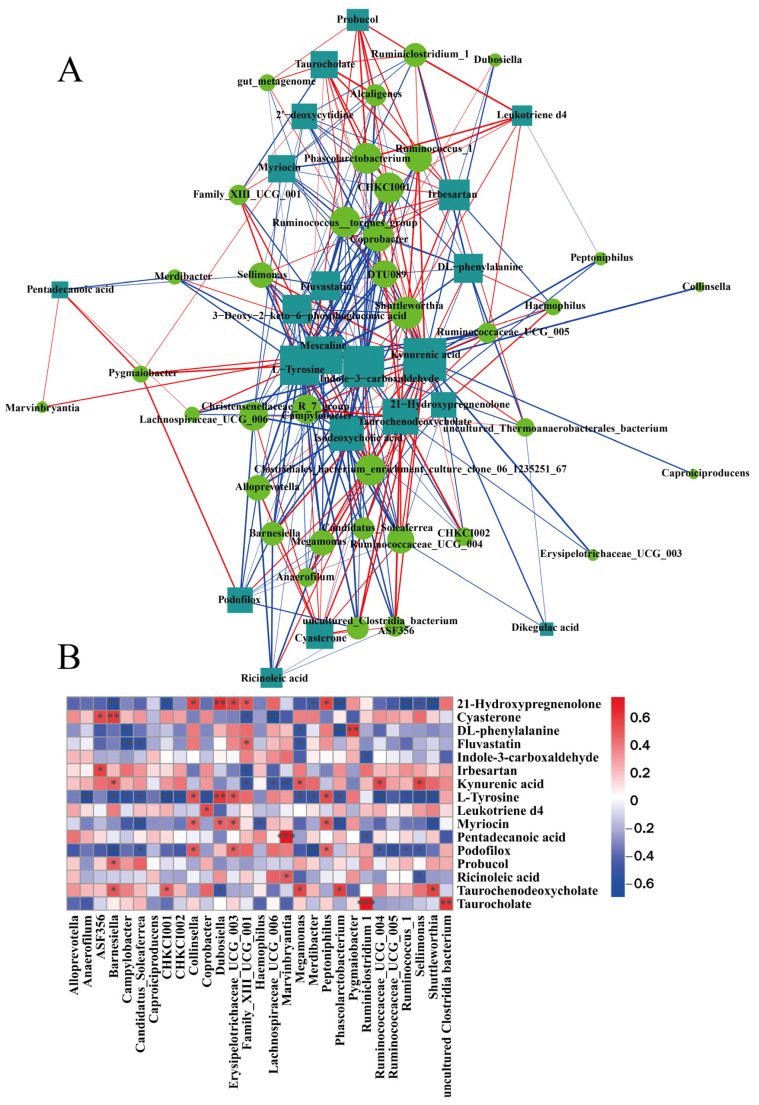
(**A**) Network diagram of Spearman’s correlation analyses for significantly different flora and significantly different metabolites. Genera with different significance levels are indicated by circles, and metabolites with different significance levels are shown by rectangles. The correlation between the two is denoted by the color of the line in the graph, with blue for negative correlation and red for positive correlation, and the line thickness is in proportion to the absolute value of the correlation coefficient. (**B**) Hierarchical cluster heatmap for Spearman correlation analysis of differentially significant flora (LEfSe LDA > 2 and *p* < 0.05) and differentially significant metabolites (*VIP* > 1 and *p* < 0.05). * *p* < 0.05, ** *p* < 0.01, *** *p* < 0.001. *VIP*: variable importance in the projection.

To explore the relationship between intestinal flora and fecal metabolites in mice, Spearman’s correlation analysis was used. The results indicate that *Caproiciproducens*, *Dubosiell*, *Erysipelotrichaceae_UCG_003*, *Pygmaiobacter*, *Lachnospiraceae_UCG_006*, and *Marvinbryantia* displayed a strong positive correlation with fluvastatin, a metabolite of the bile acid secretion pathway, and isodeoxycholic acid and pentadecanoic acid, and a negative correlation with primary bile acid biosynthetic metabolites, including taurochenodeoxycholate and taurocholate. *Shuttleworthia*, *Megamonas*, *Coprobacter*, *Barnesiella*, *Campylobacter*, *CHKCI001*, and *Anaerofilum* were positively correlated with kynurenic acid and leukotriene d4. However, these genera were negatively correlated with DL-phenylalanine, indole-3- carboxaldehyde, and ricinoleic acid. In brief, the addition of LBO was able to regulate the structure of gut microbiota and significantly altered fecal metabolites (Figure 6A,B). It is thus clear that LBO alleviated the HFD-induced intestinal flora imbalance and significantly altered the fecal metabolic profile.

## 4. Discussion 

NAFLD is recognized as the primary contributor to chronic hepatopathy worldwide and has attracted widespread public health attention. Currently, the global prevalence of NAFLD has reached 25.2% and is expected to be as high as 33.5% by 2030 [25]. The increasingly close association between intestinal flora and NAFLD at both observational and mechanistic levels has allowed using intestinal flora as biomarkers for the early diagnosis of NAFLD [26]. The composition of the gut microbiota was found to be involved in the occurrence of NAFLD. There is evidence that the dysbiosis of the intestinal flora leads to the destruction of tight junctions of the intestinal epithelium and transits bacteria and toxins through the portal vein to the liver, leading to hepatic lipid deposition and inflammation [27]. It was found that feeding the HFD to mice for eight weeks causes significantly structural and compositional alterations in the intestinal flora, leading to the emergence of NAFLD [28]. Firmicutes and Bacteroidetes are important groups of flora in the intestine. It has been observed that a higher abundance of Firmicutes and lower abundance of Bacteroidetes promote the degradation of carbohydrates [29]. LBO increases the proportion of Firmicutes and decreases the proportion of Bacteroidetes. Prebiotics such as oligofructose and inulin have the same effect [30]. *Lactobacillus* is a probiotic that has been shown to have a protective effect against metabolic diseases [31]. It has been found that *Lactobacillus* can improve liver steatosis and reduce the hepatic inflammatory response in HFD mice, which has potential therapeutic effects on NAFLD [32]. Interestingly, the addition of LBO increased the proportion of *Lactobacillus* in HFD mice. In addition, *Allobaculum*, *Dubosiella*, and *Eubacterium* were the dominant flora in the LBO group. There was a positive effect of *Allobaculum* on tryptophan metabolism, which may alleviate NAFLD development in mice [33]. *Dubosiella* has been proven to be negatively related to serum ALT concentrations, which is consistent with the present study. The increased abundance of this bacterium was able to alleviate liver damage and reduce the inflammatory response of the liver [34]. According to a previous study, *Eubacterium* is a 7α-dehydroxylase-producing bacterium, and it plays an active role in bile acid metabolism [25,35]. When bile acids entered the intestine, they were catabolized by *Eubacterium* into secondary bile acids via hydrolysis reactions [36]. The decrease in *Eubacterium* abundance caused a decrease in secondary bile acid levels, suggesting that the alterations of intestinal flora are associated with disturbances of the bile acid metabolism in NAFLD [37]. Naringin can regulate the biosynthesis of bile acid by changing *Eubacterium* abundance, which can reduce cholesterol levels [38]. Thus, LBO, at least in part, alleviated liver injury to some extent by restoring the intestinal flora. In addition to the alterations in gut flora, its metabolites may also affect the liver.

Alterations in the composition of the gut microbiota may be accompanied by shifts in intestinal metabolites. Furthermore, recent studies indicated that the development of metabolic syndrome is also relevant to gut microbiota dysbiosis [39]. To investigate the role of LBO on the metabolism of HFD mice, the metabolite content in feces was measured. In the present study, it was found that the intestinal metabolites in HFD mice were significantly modified. Bile acids (BAs) are essential metabolic regulators in the intestine–hepatic axis [40]. Fecal metabolomics showed significant alterations in the levels of taurocholate and taurochenodeoxycholate, which are bile acid metabolites that contribute to abnormal bile acid metabolism and NAFLD. In hepatocytes, cholesterol is engaged in the biosynthesis of primary bile acids, which further produces primary conjugated bile acids such as taurocholate and taurochenodeoxycholate [41]. After ingestion, bile acids can be released to the duodenum, where they are further fermented by intestinal microorganisms into secondary bile acids, affecting the absorption of nutrients [42]. Previously, it was reported that BA metabolite levels are dramatically increased in patients with NAFLD due to a reduced conversion of primary to secondary BAs [36]. Bile acids can be effectively captured and eliminated by the liver in a healthy state via hepatic portal circulation. When hepatic damage occurs, it is unable to adequately remove bile acids, contributing to higher levels of bile acids in circulation [43]. Inulin was able to reduce taurocholate levels and improve impaired lipid metabolism in NAFLD mice, and it enhanced the excretion of bile acids [44]. After adding LBO to the diet of mice with HFD, fecal metabolomics indicated a reversal of bile acid metabolite levels. LBO may improve hepatic lipid accumulation by reducing bile acid levels. Tryptophan metabolism has been found to be a critical pathway of the metabolism in hepatic inflammatory infiltration and is related to the course of NAFLD [45]. The kynurenine pathway is universally accepted to metabolize more than 95% of tryptophan, of which kynurenic acid is one of the metabolites. Kynurenic acid levels are closely correlated with the emergence of obesity and metabolic diseases. One study showed higher concentrations of kynurenic acid in the obese population [46]. Elevated levels of kynurenic acid were reversed with treatment, and liver damage was alleviated, which is in accordance with the results of this study [47]. Hydroxymethylglutaryl coenzyme A (HMG-CoA) is the rate-limiting enzyme in cholesterol biosynthesis, and fluvastatin is an inhibitor of HMG-CoA reductase [48]. An increase in fluvastatin levels can inhibit steatosis and steatohepatitis [49]. Besides the above metabolic pathways, significantly different metabolites between HFD and LBO were related to other metabolic pathways, such as tyrosine metabolism [50], arachidonic acid metabolism [51], and thiamine metabolism [52], which were associated with liver injury. These altered metabolic pathways predicted that the LBO treatment could exert a therapeutic effect by improving intestinal flora and metabolite disturbances in HFD-induced mice.

The histological features’ scoring system can be used to evaluate the pathology of the liver (Kleiner et al., 2005). LBO was found to significantly alleviate HFD-induced liver injury and hepatic steatosis by liver histological observation and histological scoring. The above phenomena indicate that LBO can significantly reverse hepatic steatosis. According to the experimental results, it is speculated that the alleviating effect of LBO on hepatic steatosis may be achieved via the regulation of intestinal flora disorders. At the same time, LBO altered intestinal metabolites that were associated with bile acid, cholesterol, and tryptophan metabolic pathways. The modulation of HFD-induced gut microbial dysbiosis using LBO may be a safe and effective strategy to alleviate hepatic steatosis and liver injury. 

## 5. Conclusions

In conclusion, the results prove the protective effect of LBO on the liver, i.e., by reducing HFD-induced lipid deposition in the liver and alleviating the process of hepatic steatosis. The mechanism of action may be that LBO modulates the structural and compositional aspects of the gut microbiota and elevates the proportion of beneficial bacteria. Moreover, LBO also affects metabolites associated with the cholesterol metabolism, bile acid metabolism, and tryptophan metabolic pathways. This research will contribute to the clinical application of LBO and its use in the production of functional foods. 

## Figures and Tables

**Table 1:** Changes in the degree of hydrolysis of LBO during simulated digestion.

Simulates Digestion	Time (h)	Degree of Hydrolysis (%)
	2	0.022 ± 0.009 ^a^
Simulates digestion in the stomach	4	0.031 ± 0.004 ^a^
	6	0.018 ± 0.004 ^a^
	2	0.039 ± 0.007 ^b^
Simulates digestion in the small intestine	4	0.022 ± 0.003 ^b^
	6	0.035 ± 0.009 ^b^

Data are shown as the mean ± SD; n = 3. Significant differences (*p* < 0.05) are signified with disparate letters. LBO: *Lycium barbarum* oligosaccharide.

## Data Availability

Data is contained within the article.
